# A Metal-Organic Framework-Based Colorimetric Sensor Array for Transcutaneous CO_2_ Monitoring via Lensless Imaging

**DOI:** 10.3390/bios14110516

**Published:** 2024-10-22

**Authors:** Syed Saad Ahmed, Jingjing Yu, Wei Ding, Sabyasachi Ghosh, David Brumels, Songxin Tan, Laxmi Raj Jaishi, Amirhossein Amjad, Xiaojun Xian

**Affiliations:** 1 McComish Department of Electrical Engineering and Computer Science, Jerome J. Lohr College of Engineering, South Dakota State University, Brookings, SD 57007, USA; syed.ahmed@jacks.sdstate.edu (S.S.A.); jingjing.yu@fjut.edu.cn (J.Y.);; 2 College of Ecological Environment and Urban Construction, Fujian University of Technology, Fuzhou 350118, China

**Keywords:** transcutaneous gas, carbon dioxide, colorimetric sensor, MOFs, CMOS Imager

## Abstract

Transcutaneous carbon dioxide (TcPCO2) monitoring provides a non-invasive alternative to measuring arterial carbon dioxide (PaCO2), making it valuable for various applications, such as sleep diagnostics and neonatal care. However, traditional transcutaneous monitors are bulky, expensive, and pose risks such as skin burns. To address these limitations, we have introduced a compact, cost-effective CMOS imager-based sensor for TcPCO2 detection by utilizing colorimetric reactions with metal–organic framework (MOF)-based nano-hybrid materials. The sensor, with a colorimetric sensing array fabricated on an ultrathin PDMS membrane and then adhered to the CMOS imager surface, can record real-time sensing data through image processing without the need for additional optical components, which significantly reduces the sensor’s size. Our system shows impressive sensitivity and selectivity, with a low detection limit of 26 ppm, a broad detection range of 0–2% CO_2_, and strong resistance to interference from common skin gases. Feasibility tests on human subjects demonstrate the potential of this MOF-CMOS imager-based colorimetric sensor for clinical applications. Additionally, its compact design and responsiveness make it suitable for sports and exercise settings, offering valuable insights into respiratory function and performance. The sensing system’s compact size, low cost, and reversible and highly sensitive TcPCO2 monitoring capability make it ideal for integration into wearable devices for remote health tracking.

## 1. Introduction

Carbon dioxide (CO_2_) plays multiple roles in the human body, such as regulating blood pH, driving respiratory, and lowering the oxygen affinity of hemoglobin to facilitate oxygen unloading [1,2]. Monitoring CO_2_ from the human body is important for assessing human health.CO_2_ levels are usually monitored through breath gas, blood gas, and transcutaneous gas [3,4]. The concentrations detected by these methods show good correlations with each other [5,6,7,8,9,10]. Among these methods, arterial blood gas analysis (ABG) is the gold standard for PaCO_2_ (the arterial partial pressure of carbon dioxide) measurement, which is used in clinical settings to indicate respiratory function and ventilation [11]. However, this method requires the withdrawal of arterial blood and laboratory analysis; thus, it is invasive, expensive, painful for patients, and cannot be used for continuous and remote monitoring [11]. Transcutaneous carbon dioxide (TcPCO2) levels are closely correlated with arterial carbon dioxide (PaCO2) levels, making this method valuable in various applications, including sleep diagnostics, neonatal intensive care, and exercise testing.

Current technologies for transcutaneous CO_2_ detection include a variety of sensor types, such as electrochemical sensors, luminescence-based sensors, non-dispersive infrared (NDIR) sensors, colorimetric-based sensors, and photoacoustic spectroscopy [5,7,8,12,13,14,15]. Severinghaus-type CO_2_ electrochemical sensors offer continuous, non-invasive CO_2_ monitoring with high stability, sensitivity, and quick response times. However, they require frequent calibration, maintenance, and heating of the skin, leading to potential complexity, discomfort, and time lag in detecting CO_2_ changes [16,17]. Luminescence-based CO_2_ sensors provide short response times, high sensitivity, and a broad dynamic range for detection [12,15]. However, they face challenges due to confounding factors affecting fluorescence intensity, the need for specialized high-speed instrumentation due to nanosecond fluorophore lifetimes, and a dependency on temperature, which limits their use in low-power and miniaturized devices [18]. NDIR sensors are favored for CO_2_ monitoring due to their high accuracy, stability, selectivity, wide measurement range, and fast response time [19]. However, their output is susceptible to interference from water vapor and ambient temperature, complicating transcutaneous CO_2_ measurement [6,20]. Quartz tuning fork-based photoacoustic sensors offer continuous real-time CO_2_ monitoring with a compact size and low cost, but their accuracy can be affected by temperature, humidity, and motion [21]. While each method has its own strengths and limitations in terms of sensitivity, cost, and ease of use, commercially available transcutaneous CO_2_ devices primarily rely on electrochemical sensors. However, these commercial devices are costly, around $20,000 [4], and require heating of the skin to enhance CO_2_ diffusion, which can lead to discomfort and potential skin burns [22]. Therefore, developing technologies that are low cost and do not need to increase skin temperature can enable point-of-care or home-use transcutaneous CO_2_ monitors. Among these technologies, colorimetric sensors show some unique advantages, such as being cost-effective, miniature, easy to fabricate, and suitable for multiplexed monitoring [5,23,24]. 

The colorimetric gas sensor is based on the colorimetric reaction between the gas and sensing indicators. The sensor’s sensitivity is not only influenced by the sensing indicator but also by the matrix [25]. A porous matrix with high surface area would be beneficial for increasing the sensor sensitivity. Metal–organic frameworks (MOFs), composed of metal ions (or metal clusters) and organic linkers, have garnered significant attention in recent years owing to their high surface area and permanent porosity [26,27]. The incorporation of MOFs in colorimetric sensors could enhance sensing performance due to multiple mechanisms. First, MOFs offer an exceptionally high surface area, which can increase the interaction with target molecules and significantly improve the sensor’s sensitivity [28]. Their tunable pore sizes allow for selective detection, enabling the precise targeting of specific chemicals. This selectivity is further enhanced by MOFs’ ability to be engineered for preferential interaction with analytes, which reduces interference from other substances and leads to more accurate measurements [29,30]. Additionally, MOFs can serve as the matrix for loading sensing probes, which significantly increases the active sites available for analyte binding, further boosting the sensor’s efficiency [31,32,33]. The versatility of MOFs also allows them to be tailored for use in developing advanced, reliable colorimetric sensors for a broad range of applications [26,34].

In this work, we report on a compact, cost-effective CMOS imager-based sensing system designed to detect transcutaneous CO_2_ through colorimetric chemical reactions involving CO_2_ and MOF-71-based nano-hybrid sensing materials. By printing the nano-hybrid sensing materials onto an ultrathin PDMS membrane (25 μm thickness) to create a colorimetric sensing spot array and adhering this membrane to the CMOS imager surface, we have developed a compact colorimetric sensor capable of real-time signal recording via image processing. This innovative sensor design eliminates the need for additional optical components and significantly reduces the size of the sensing system. Moreover, the sensor’s high sensitivity enables detection at skin temperature without heating, thereby preventing skin burns and conserving power. Our system demonstrates high sensitivity and good selectivity, with a low detection limit of 26 ppm, a wide detection range of 0–2% CO_2_, and strong selectivity against common interferents in skin gases. Feasibility tests on human subjects highlight the potential of this MOF-CMOS imager-based colorimetric sensor for clinical applications. Our tests have validated the system’s ability to provide the reversible and highly sensitive monitoring of transcutaneous carbon dioxide (TcPCO2), demonstrating its potential for seamless integration into wearable devices. This capability makes it an ideal solution for the continuous monitoring of patients with conditions such as COVID-19, sleep apnea, and chronic obstructive pulmonary disease (COPD). The sensor’s accuracy and responsiveness allow it to detect even subtle changes in CO_2_ levels, which is crucial for managing these respiratory conditions. Additionally, the system’s versatility extends beyond clinical applications. Its compact design and high sensitivity make it suitable for use in sports and exercise settings, where the real-time monitoring of CO_2_ levels can provide valuable insights into an athlete’s respiratory function and overall performance.

## 2. Materials and Methods

### 2.1. Materials

M-Cresol purple, tetrabutylammonium hydroxide (25% in methanol), glycine, ethanol (99.5%), cobalt nitrate hexahydrate (Co (NO_3_)_2_·6H_2_O), 1,4-benzene dicarboxylic acid (H_2_BDC), and N, N-dimethylformamide (DMF) were purchased from Sigma-Aldrich (St. Louis, MO, USA). The polydimethylsiloxane (PDMS) membrane (thickness: 0.025 mm; Manufacturer: JIAWANSHUN, Nanyang, China) was purchased from Amazon. Pure CO_2_ and clean air were purchased from Matheson Inc. (Sioux Falls, SD, USA).

### 2.2. Synthesis of MOF-71

**Figure 1:**
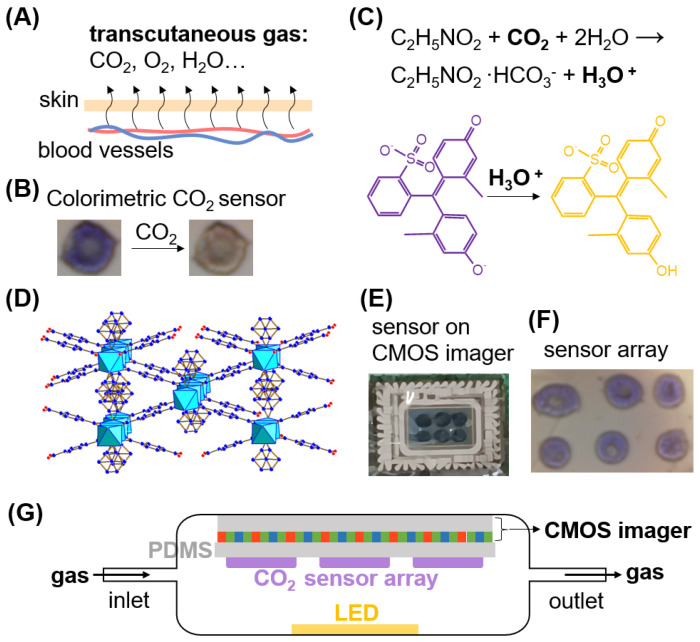
Principle of the colorimetric gas sensor for transcutaneous gas monitoring. (**A**) Schematic of the diffusion of blood gas through the skin. (**B**) Images showing the typical color change in the sensing spot in the array when exposed to CO_2_. (**C**) The chemical reactions that lead to the colorimetric detection of CO_2_. (**D**) The structure of Co-MOF-71. The light blue octahedrons, the red balls, the dark blue balls, and the green balls represent Co(II) centers, oxygen atoms, carbon atoms, and hydrogen atoms, respectively. (**E**) The photo shows the PDMS membrane with the sensor array attached to the CMOS imager, captured using a phone camera. (**F**) Lensless image of the CO_2_ sensor array captured by the CMOS imager with its pixels. (**G**) Schematic of the lensless colorimetric CO_2_ sensing system.

The cobalt-based MOF-71 was synthesized by a solvothermal reaction according to the reported method [35,36]. First, 0.75 g of Co (NO_3_)_2_·6H_2_O and 0.42 g of H_2_BDC were dissolved in 12 mL of ethanol and 48 mL of DMF. The mixture was transferred to a Teflon-lined autoclave where the solution was heated at 100 °C for 12 h. Once the autoclave was cooled down to room temperature, a pink/purple crystalline powder was obtained by centrifugation at the rate of 500 rpm. Finally, the precipitate was washed three times with the DMF to remove the impurities. The structure of MOF-71 is shown in Figure 1D.

### 2.3. Preparation of Sensor Chip

Preparation of the colorimetric sensing solution of CO_2_ indicator: The colorimetric sensing solution was prepared by dissolving 16.7 mg M-cresol purple and 100 mg glycine into 12.3 mL ethanol and 3 mL tetrabutylammonium hydroxide (25% in methanol). Furthermore, 1 mL of colorimetric sensing solution was mixed with 10 mg of MOF-71 for sensor fabrication.

Fabrication of sensor chip: The sensing solution with or without MOF was dispensed on the PDMS membrane through a Fisnar f-4200 N benchtop robot (Germantown, WI, USA) with a 0.33 mm needle and a dispense volume of 1 µL. The 2 × 3 sensing spots array was dispensed on the PDMS membrane and then dried in a vacuum for ~15 min before the test. The dried sensor chip (PDMS membrane with 2 × 3 sensing spots array) was then adhered to the surface of the CMOS imager (Logitech (Suzhou, China) C525 Webcam with the lens removed), as shown in Figure 1E.

### 2.4. Characterization of Sensing Material

The MOF-71 with and without colorimetric sensing material (cresol purple, glycine, and tetrabutylammonium hydroxide) were characterized. The morphologies were analyzed using scanning electron microscopy (S-4700, FESEM, Hitachi, Tokyo, Japan). X-ray diffraction (XRD) was performed with a Rigaku SmartLab diffractometer (Tokyo, Japan), and Fourier transform infrared spectroscopy (FTIR) was conducted using a Nicolet 6700 (Thermo Scientific, Waltham, MA, USA).

### 2.5. Gas Sample Preparation and Skin Gas Collection

Calibration gas preparation: CO_2_ gas of 100 ppm–20,000 ppm (2%) was prepared by diluting pure CO_2_ with humid air into sampling bags. The humid air was prepared by adding water to the bag filled with clean air. The humidity of the CO_2_ samples was maintained at 85%RH level to simulate the condition of skin gas. Clean air with the same level of humidity was used as purging gas for sensor tests.

Skin gas collection: Before skin gas collection, subjects were required to wash their hands and arms with fresh water and then dry them with wipers. The hand was inserted in the sampling bag and sealed. The gas in the bag was pulled out, and 10 L of clean air was pushed in the bag. After 30 min, the collected gas was placed in another clean bag for testing. The CO_2_ concentration and humidity of the collected skin were checked with a commercial CO_2_ m (Telaire-7001 CO_2_ monitor, Miesbach, Germany) and Sensirion SHT3x humidity sensor (Stäfa, Switzerland). The skin gas of subjects at rest and doing light exercise (such as sit-ups or jogging in place) was collected. 

### 2.6. Gas Test Method

The sensing chip was attached to the top of a CMOS imager. Then, the gas chamber and LED were assembled with the CMOS imager to build the detection device (Figure 1). Clean air and sampling gas of different CO_2_ concentrations or skin gases were introduced into the gas chamber at 0.5 L/min alternately. LED was used for illuminating the sensing chip. The color of the sensing chip was monitored by a CMOS imager. A MATLAB R2021b program was used to identify the sensor and process the captured images in real time. 

### 2.7. Human Subjects

Four healthy subjects without regular medication and respiratory diseases were recruited to participate in the study. The study was approved by the IRB of South Dakota State University (IRB reference protocol number #001967). All the subjects participated in the study voluntarily and signed consent forms before the tests. 

## 3. Results and Discussion

### 3.1. Sensing Principle of Colorimetric CO_2_ Detection via Lensless Imaging

Transcutaneous monitoring is a non-invasive technique that measures the partial pressure of gases, such as oxygen and carbon dioxide, through the skin. This method relies on the natural diffusion of these gases from the underlying blood vessels through the skin (Figure 1A), which acts as a semi-permeable membrane [12,37]. Numerous studies have demonstrated a strong correlation between the levels of CO_2_ in blood gases and transcutaneous gasses [7,9,10]. Therefore, by noninvasively detecting transcutaneous CO_2_, the level of CO_2_ in blood gases can be accurately assessed. Unlike the conventional Stow–Severinghaus method, which determines transcutaneous CO_2_ based on the linear relationship between pH and the logarithm of PCO_2_ [12,38,39], our sensor detects transcutaneous CO_2_ by utilizing the CO_2_-induced color change in a pH indicator (Figure 1B). The colorimetric detection is based on a carbamate reaction and the subsequent pH change in a pH indicator. Cresol purple, the pH indicator, is combined with hexadecyltrimethylammonium hydroxide and glycine to detect CO_2_ (Figure 1C). Hexadecyltrimethylammonium hydroxide adjusts the pH of the sensing solution to the appropriate initial level to maximize the range of the color change. When glycine reacts with CO_2_, it forms a carbamate, releasing a proton in the process. This reaction is fast, high-yield, and reversible. The released proton then reacts with the pH indicator, causing its color to shift from purple to yellow (Figure 1B,C). As the CO_2_ concentration increases, more protons are produced, leading to a greater color change from purple to yellow. Thus, the extent of the color change can be used to determine the CO_2_ concentration.

Without an appropriate matrix to load and support the colorimetric sensing probes, the active sites for CO_2_ adsorption and the reaction are significantly limited, resulting in the lower sensitivity of the sensor. MOFs offer a solution to this challenge due to their highly porous structure and large surface area. These characteristics enable MOFs to effectively load and distribute the colorimetric sensing probes, thereby increasing the number of active sites available for CO_2_ interaction. Specifically, MOF-71 (Figure 1D), a well-studied MOF that can be synthesized through a simple one-step hydrothermal method [35,36,40], has been utilized to enhance the performance of the chemical sensors [41]. By incorporating MOF-71 with the colorimetric indicator, the sensor’s sensitivity can be improved. The porous nature of MOF-71 allows for a larger amount of CO_2_ to be adsorbed onto the surface, facilitating more efficient reactions between CO_2_ and the indicator. This increased interaction between the CO_2_ molecules and the sensing probes leads to a more pronounced color change, thereby enhancing the sensor’s sensitivity and selectivity. Moreover, the uniform pore structure of MOF-71 contributes to a more consistent and reliable sensor response, as it ensures that CO_2_ molecules are uniformly distributed across the sensing surface. This homogeneity not only boosts the sensitivity but also improves the selectivity of the sensor by minimizing interference from other gases. The integration of MOF-71 into the colorimetric sensor significantly enhances its ability to detect CO_2_ with greater accuracy and precision. 

Traditionally, colorimetric sensing arrays are printed on a substrate and then imaged using an optical system [42,43,44]. However, this approach poses challenges for miniaturization due to the large size of each printed sensing element, the need for bulky optical components, and the requirement for a specific focal distance. To overcome these limitations, we transformed a conventional CMOS imager into a lensless colorimetric sensor chip (Figure 1E). This was achieved by directly coating a colorimetric microdroplet array onto an ultra-thin PDMS membrane, just 25 μm thick, and then adhering this coated membrane onto the surface of the CMOS imager. As the solvent evaporated, millimeter-scale solid colorimetric sensing spots formed on the PDMS membrane. The solution coating and solvent drying processes ensure that the sensing spots are firmly adhered to the membrane. The ultra-thin and transparent PDMS membrane maximizes the transmission of light through the sensing spots, allowing the underlying photodetectors in the CMOS pixels to capture high-resolution images of the sensing array. Due to the flexibility and thinness of the PDMS membrane, it adheres seamlessly to the surface of the CMOS imager, enabling effective lensless imaging by the underlying pixels. Additionally, the hydrophobic nature of PDMS minimizes the impact of humidity on the colorimetric detection process [6]. Because the sensing spots are closely attached to the CMOS imager surface (Figure 1E,G), they can be clearly imaged by the micro-optic array (pixels) without the need for lenses (Figure 1F). This design significantly reduces the size and complexity of the sensor, making it much more compact and suitable for wearable or portable applications, as no focal distance is required. By tracking the color changes (in the red, green, and blue channels) of the sensing spots through images captured by the CMOS imager and processed using MATLAB, the transcutaneous CO_2_ levels can be quantified. This innovative approach provides a more efficient and scalable solution for real-time colorimetric sensing, particularly in applications where size and portability are critical. 

Using a sensing array can reduce variation and enhance the reproducibility of transcutaneous CO_2_ detection by enabling simultaneous measurements across multiple sensing spots. This array-based approach allows for the averaging of data from various spots, which minimizes the impact of localized anomalies or inconsistencies in individual sensors, resulting in a more stable and reliable overall signal. The redundancy provided by the array ensures that any outlier readings can be easily identified and excluded, further improving the accuracy and reproducibility of the detection process. Additionally, the sensor array offers the potential to detect multiple transcutaneous gases simultaneously when integrated with other sensing probes. This capability will be explored in our future work to provide a more comprehensive monitoring solution for multiplexed transcutaneous gas detection. 

### 3.2. Characteristics of the MOF-71

**Figure 2:**
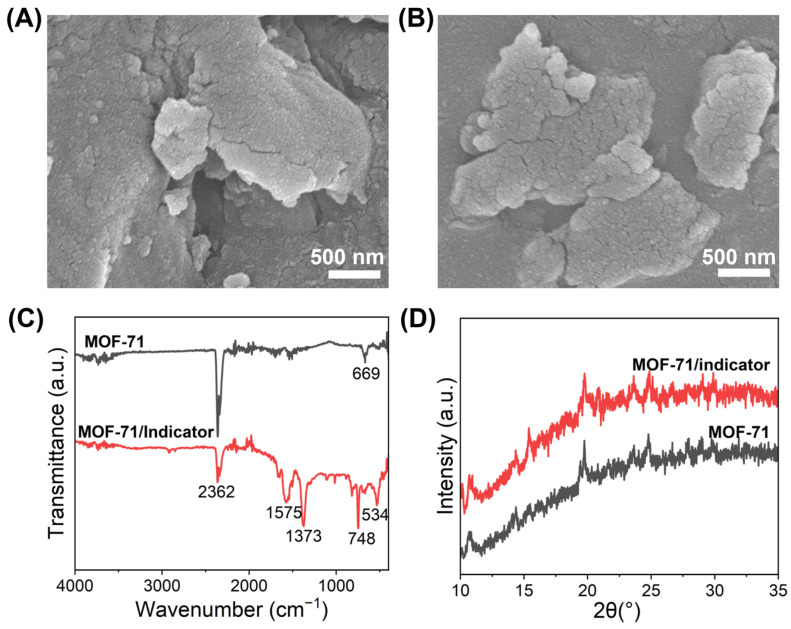
Characteristics of MOF-71 with and without colorimetric CO_2_ indicator. (**A**) SEM images of MOF-71. (**B**) SEM images of MOF-71 with CO_2_ indicator. (**C**) FTIR spectra of MOF-71 with and without CO_2_ indicator. (**D**) XRD patterns of MOF-71 with and without CO_2_ indicator.

The SEM images of MOF-71 and the mixture of MOF-71 with the CO_2_ indicator are shown in Figure 2A,B, respectively. MOF-71 in Figure 2A,B appears to be composed of aggregates of sub-micron particles. The surface morphology did not change after being mixed with the CO_2_ indicator, indicating the stability of the MOF that can be used as a matrix for the CO_2_ indicator. FTIR characterization was also performed as shown in Figure 2C,D. The peak of 669 cm^−1^ in Figure 2C is attributed to the Co-O vibration [45], confirming the formation of cobalt hydroxide in MOF-71 material. After mixing with the CO_2_ indicator solution, new characteristic peaks appear, including at 1575 cm^−1^, 1373 cm^−1^, 748 cm^−1^, and 534 cm^−1^. The band at 1575 cm^−1^ corresponds to the symmetric C-O vibrations, while the bands of 1373 cm^−1^ and 748 cm^−1^ are attributed to the asymmetric stretching of C-O and the bending vibration of C-H, respectively. Compared to the pure MOF-71, the peak of cobalt hydroxide in the MOF-71/CO_2_ indicator material shifted from 669 to 534 cm^−1^. The powder X-ray diffraction (XRD) patterns of the MOF material and the MOF mixed with the CO_2_ indicator show similar peaks (Figure 2D) to the previously reported literature of MOF-71 [35]. Based on the XPS data reported in the studies, the characteristic peaks confirm the presence of Co^2+^ in MOF-71 [35,36,46,47]. According to the literature, MOF-71 has an average pore diameter of 66.91 nm and a surface area of 4.57 m^2^/g [48]. This large surface area offers numerous binding sites for CO_2_ adsorption, which is crucial for enhancing the sensor’s sensitivity.

### 3.3. Analytical Performance of MOF-Based Colorimetric CO_2_ Sensor Array

**Figure 3:**
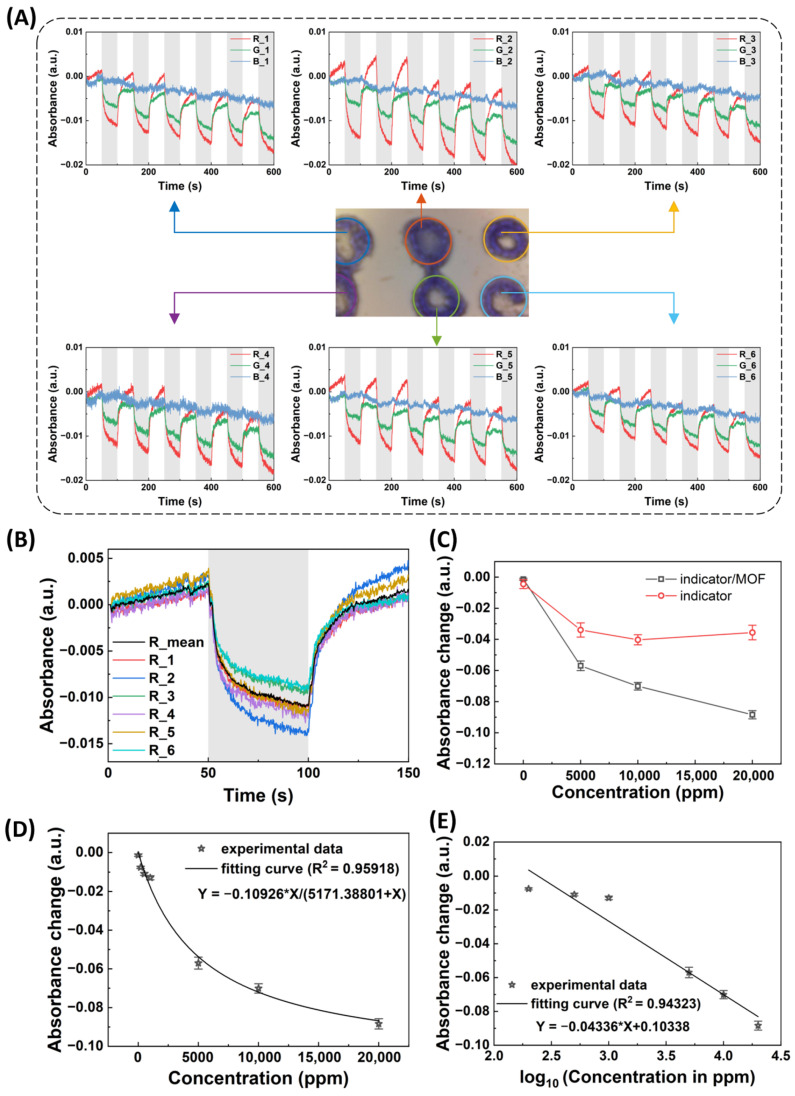
Sensing performance of the MOF-based colorimetric sensor array for CO_2_ detection. (**A**) Real-time absorbance changes in each sensing spot in the sensor array when exposed to clean air (blank area) and 1000 ppm CO_2_ (gray shade area) gas alternately for six cycles. Absorbance was calculated with intensity from the CMOS imager. R, G, and B represent the red, green, and blue channels of the CMOS imager, respectively. (**B**) Comparison of the absorbance (R channel) of each sensing spot in the sensor array and their average absorbance. (**C**) Response of the sensor arrays with and without MOF-71 to different concentrations of CO_2_ (R channel). Absorbance change was calculated from absorbance difference before and after the CO_2_ exposure. (**D**) Calibration plot of the sensor array for CO_2_ detection in the range of 0 to 20,000 ppm. (**E**) Calibration plot of the sensor with the CO_2_ concentration in log scale.

The sensor response to CO_2_ was evaluated by exposing the sensor array to air and CO_2_ alternately for six cycles (Figure 3A). The absorbance was calculated with the intensity from the CMOS imager by the following equation:

$$Absorbance=-{log}_{10}\frac{intensity}{initial\;intensity}$$

where intensity is captured by a CMOS imager and processed with a MATLAB R2021b program. The initial intensity is the intensity obtained from the first image frame. The image data consists of three channels: red (R), green (G), and blue (B). The intensity from each channel was used to calculate the absorbance specific to that channel.

Figure 3A shows the real-time absorbance change in the sensor array. The absorbance changes in different sensing spots in the sensor array were similar to each other. The sensor array showed reversible sensing behavior for CO_2_ detection with a response time and recovery time of ~50 s. The absorbance of R and G channels decreased under CO_2_ exposure, which was due to the color change from purple to yellow. The R channel showed a greater absorbance change compared to the G channel, making the R channel signal the preferred choice for evaluating sensor performance. The data in Figure 3A show baseline drifts. The optical signal drift is primarily caused by the warm-up effect of the LED as its performance is temperature sensitive. Current flow generates heat in the LED chip, which can alter the output spectrum, chromaticity, and intensity. To mitigate baseline drift, we can enhance the sensing platform design by either (1) integrating a thermistor and applying signal compensation algorithms to correct for temperature effects or (2) introducing a warm-up period to achieve thermal stability before measurement. Figure 3B compares the R absorbance of different sensing spots in the CO_2_ sensor array. The absorbance change caused by CO_2_ varied across different sensing spots in the sensor array, with a difference of approximately 17%, which can be attributed to the non-uniform coating from the dispenser. This issue can be mitigated by employing more precise coating techniques, such as 3D printing. This variation also highlighted the importance of averaging the response from each sensing spot in the sensor array to enhance the consistency and reproducibility of CO_2_ detection. We calculated the average absorbance across six sensing spots in the sensor array, represented by the black curve in Figure 3B. The noise level of the average absorbance (black curve, noise level of ~1.85 × 10^−4^) was much less than the noise level of the single sensing spots (other curves in Figure 3B, noise level ~3.17 × 10^−4^). Thus, the average signal from each sensing spot in the sensor array can effectively decrease the noise level, leading to the detection limit being improved by ~1.7-times.

To assess the impact of MOF-71 on sensor performance, we conducted similar tests as those in Figure 3A on sensors both with and without MOF-71. CO_2_ with concentrations ranging from 5000 ppm to 20,000 ppm was used to evaluate sensor sensitivity. As shown in Figure 3C, the sensor incorporating MOF-71 exhibited higher sensitivity (sensitivity doubled) than the sensor without MOF-71, which is due to the high surface area of MOF-71 for loading the CO_2_ indicator. These results demonstrate that MOF-71 can significantly enhance sensor performance for CO_2_ detection. MOFs with high CO_2_ adsorption have pores that are compatible with CO_2_’s kinetic diameter and contain polar sites to enhance adsorption [49]. We speculate that MOF-71’s pore structure and polar functional groups create an optimal environment for CO_2_ capture, making it a strong candidate for sensing applications. Further investigation is needed to confirm this interaction and its efficiency. 

We further evaluated the sensor’s sensitivity to CO_2_ at a concentration lower than 1000 ppm, as shown in Figure 3D. The results indicate that the sensor can detect CO_2_ with a concentration lower than 200 ppm. Assuming the detectable sensor response is three times the absorbance noise (signal-to-noise ratio of 3), the theoretical detection limit of the CO_2_ sensor array is ~26 ppm. This detection limit is enough for detecting the transcutaneous CO_2_. We also presented a calibration plot with CO_2_ concentrations in the log scale (Figure 3E). A linear regression line was obtained with an R square of 0.9433, as shown in Figure 3E. This indicates that the CO_2_ sensor array can be calibrated with a simple procedure.

**Figure 4:**
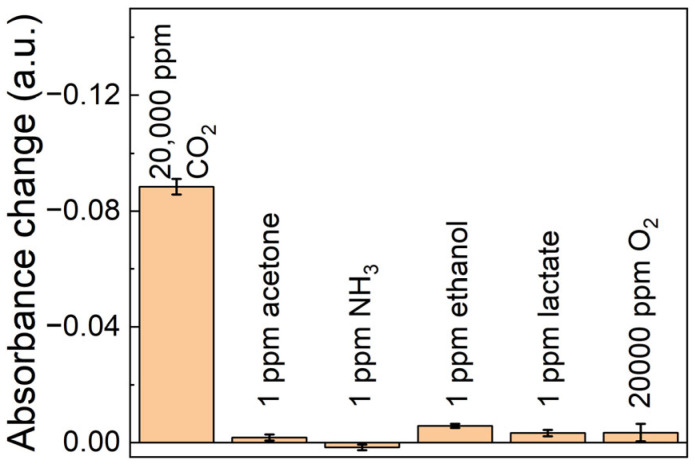
Cross-sensitivity of the MOF-based colorimetric CO_2_ sensor array.

We further evaluated the cross-sensitivity of the sensor by comparing its response to various gases, including 1 ppm acetone, 1 ppm NH_3_, 1 ppm ethanol, 1 ppm lactate, and 20,000 ppm oxygen. These gases were selected because they represent the primary transcutaneous gases typically found in skin emissions. The concentrations used in this study were chosen to reflect their typical levels in skin gases, as reported in previous studies [50,51]. The results, presented in Figure 4, demonstrate that our sensor exhibits strong selectivity for CO_2_, showing minimal interference from the other primary transcutaneous gases tested. This indicates that the sensor is highly effective at distinguishing CO_2_ from other gases commonly present in the skin, thereby ensuring CO_2_ measurements without significant cross-sensitivity. This selectivity is essential for maintaining the precision of transcutaneous CO_2_ monitoring in real-world applications.

### 3.4. Transcutaneous CO_2_ Measurement with Human Subjects

**Figure 5:**
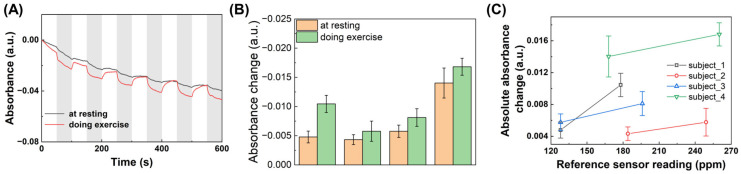
Transcutaneous CO_2_ measurement with human subjects. (**A**) Real-time absorbance change curve of the MOF-based colorimetric CO_2_ sensor array exposed to air and skin gas alternately. The blank areas indicate the air purging, and the gray shades indicate the exposure of skin gas. The skin gas was collected from a human subject while resting or doing exercise. The collection time of skin gas is 30 min. (**B**) The colorimetric sensor response to skin gas collected from different human subjects at resting and doing exercise. (**C**) Correlation between the readings from the MOF-based colorimetric CO_2_ sensor array and commercial CO_2_ sensor.

The feasibility of using the MOF-based colorimetric CO_2_ sensor array for transcutaneous CO_2_ detection in real-world applications was demonstrated through testing on four human subjects. Skin gas samples were collected from each subject using Tedlar bags and then analyzed using both the MOF-based colorimetric CO_2_ sensor array and a commercial CO_2_ sensor. In the experiment, a 10-liter Tedlar bag was used to cover one hand of each subject, and the bag was sealed with tape to prevent gas leakage. After the bag was emptied, it was filled with clean air. The subject then either remained at rest or engaged in light physical activity (such as sit-ups or jogging in place) for 30 min. Following this period, the collected transcutaneous gas samples were analyzed using both the MOF-based colorimetric CO_2_ sensor array and the commercial CO_2_ sensor. The results showed that skin gas collected during exercise exhibited a higher CO_2_ response compared to samples collected at rest, indicating that the body emits more CO_2_ through the skin during physical activity. This finding, illustrated in Figure 5A,B, aligns with previous research [5,6,8,13]. Furthermore, the CO_2_ levels measured by the MOF-based colorimetric sensor array closely correlated the readings obtained from the commercial CO_2_ sensor for each subject, as shown in Figure 5C. These results from human subject testing suggest that the MOF-based colorimetric CO_2_ sensor array is a viable tool for transcutaneous CO_2_ monitoring, with potential applications in healthcare settings. For practical applications, sensor stability is crucial. The overall stability of colorimetric sensors is typically influenced by the chemical properties of the sensing materials, as well as the packaging and storage conditions. To ensure that our MOF-based sensor meets the requirements for reliable, long-term use, we will thoroughly investigate its stability over extended periods in the next phase of our research.

## 4. Conclusions

In conclusion, this study successfully demonstrated the feasibility of using an MOF-based colorimetric CO_2_ sensor array for transcutaneous CO_2_ detection in real-world applications. The developed sensor, based on MOF-71, a pH indicator, and a CMOS imager, exhibits high sensitivity, a low detection limit of 26 ppm, a wide detection range of 0–2% CO_2_, and excellent selectivity. By leveraging the unique properties of MOF-71, we were able to enhance the sensor’s performance, enabling improved transcutaneous CO_2_ detection. The testing on human subjects further validated the sensor’s capabilities, showing a strong correlation with a commercial CO_2_ sensor, confirming its ability to monitor the average CO_2_ levels during different physical states, such as rest and exercise, and demonstrating its potential for the post hoc analysis of human physical states. The results indicate that this innovative sensor array can effectively distinguish CO_2_ from other transcutaneous gases, minimizing cross-sensitivity and ensuring precise measurements. The study also highlights the potential of the MOF-based sensor array to be integrated into wearable or portable devices, offering a non-invasive solution for continuous CO_2_ monitoring in healthcare settings. Future work will aim to enhance the sensor’s capabilities for the simultaneous detection of multiple gases and improve its performance by reducing baseline drifting, thereby broadening its potential applications. Overall, this research paves the way for the development of advanced, miniaturized sensors that can provide critical real-time data for medical diagnostics and personalized health monitoring.

## Data Availability

The original contributions presented in the study are included in the article, further inquiries can be directed to the corresponding author.
